# Response of enzyme activities and microbial communities to soil amendment with sugar alcohols

**DOI:** 10.1002/mbo3.355

**Published:** 2016-03-23

**Authors:** Huili Yu, Peng Si, Wei Shao, Xiansheng Qiao, Xiaojing Yang, Dengtao Gao, Zhiqiang Wang

**Affiliations:** ^1^ Zhengzhou Fruit Research InstituteChinese Academy of Agricultural SciencesZhengzhouHenan450000China

**Keywords:** Biolog CLPP, enzyme activity, soil microbial community, sugar alcohols

## Abstract

Changes in microbial community structure are widely known to occur after soil amendment with low‐molecular‐weight organic compounds; however, there is little information on concurrent changes in soil microbial functional diversity and enzyme activities, especially following sorbitol and mannitol amendment. Soil microbial functional diversity and enzyme activities can be impacted by sorbitol and mannitol, which in turn can alter soil fertility and quality. The objective of this study was to investigate the effects of sorbitol and mannitol addition on microbial functional diversity and enzyme activities. The results demonstrated that sorbitol and mannitol addition altered the soil microbial community structure and improved enzyme activities. Specifically, the addition of sorbitol enhanced the community‐level physiological profile (CLPP) compared with the control, whereas the CLPP was significantly inhibited by the addition of mannitol. The results of a varimax rotated component matrix demonstrated that carbohydrates, polymers, and carboxylic acids affected the soil microbial functional structure. Additionally, we found that enzyme activities were affected by both the concentration and type of inputs. In the presence of high concentrations of sorbitol, the urease, catalase, alkaline phosphatase, *β*‐glucosidase, and *N*‐acetyl‐*β*‐d‐glucosaminidase activities were significantly increased, while invertase activity was decreased. Similarly, this increase in invertase, catalase, and alkaline phosphatase and *N*‐acetyl‐*β*‐d‐glucosaminidase activities was especially evident after mannitol addition, and urease activity was only slightly affected. In contrast, *β*‐glucosidase activity was suppressed at the highest concentration. These results indicate that microbial community diversity and enzyme activities are significantly affected by soil amendment with sorbitol and mannitol.

## Introduction

Amending soil with low‐molecular‐weight organic compounds, such as amino acids, sugars, and carboxylates can stimulate increases in carbon mineralization, microbial biomass, and the number of culturable bacteria (Nakatsu et al. 2005; Raffa et al. 2005; Chaparro et al. 2013). Broadly, these compounds are associated with root exudation, plant residues, and soil fauna excretes and may be utilized by soil microorganisms and affect the quality of the microbial community. Hopkins et al. (2008) found that soil respiration responded positively to both glucose and NH_4_Cl additions, along with associated changes in soil microbial community structure. Glucose addition has consistently been shown to cause significant shifts in microbial community structure (Falchini et al. 2003; Hoyle et al. 2008; Dungait et al. 2011), and oxalic acid supplementation has been shown to have similar effects (Landi et al. 2006). The addition of phenol and oxalate led to enhanced degradation of soil organic matter as compared to glucose and glutamate addition (Brant et al. 2006). Sugar alcohols, such as sorbitol and mannitol, are produced by microorganisms and plants, can be present at levels of 0.33 and 0.8 *μ*g·g^−1^, respectively, in olive tree rhizospheric soil, and in trace amounts in soil (Roser et al. 1994; Mechri et al. 2015), and can serve as carbohydrate reserves, storage of reducing power, translocatory compounds, and osmoprotectants (Wisselink et al. 2002; Akinterinwa et al. 2008; Liebeke et al. 2009). Additionally, sugar alcohols enhance the growth of plants, fungi, yeasts, and bacteria under stress (Stoop et al. 1996; Chaturvedi et al. 1997; Ichimura et al. 2016). However, until now, there have been no investigations of the effects of soil amendment with sugar alcohols on soil microbial functional diversity and enzyme activities.

Soil microorganisms are crucial to many ecosystem processes, promoting plant growth, nutrient cycling, soil structure, and energy flow. These functions are of great importance to the productivity of agricultural soils (Juarez et al. 2013). Yet, soil microorganisms are very sensitive to any ecosystem disturbance that rapidly alters community diversity and activity (Vallejo et al. 2010; Berthrong et al. 2013; Ouni et al. 2013). Soil microbial community properties, particularly those related to diversity and functional activity, can serve as useful predictors of the impact of substrate amendment on soil quality.

(Kızılkaya et al. 2004; Wu et al. 2014). Soil amendment with low‐molecular‐weight organic substances can change microbial activity (including microbial biomass C and metabolic quotient, qCO_2_) (Steinbeiss et al. 2009; Fischer et al. 2010; An et al. 2015) and community structure. These effects have been investigated in a number of studies using molecular profiling or similar methods, such as ^13^C tracer (Maxfield et al. 2012; Juarez et al. 2013), denaturing gradient gel electrophoresis (DGGE; Tortella et al. 2013), phospholipids fatty acid analysis (PLFA; Dungait et al. 2011; Ai et al. 2015), and quantitative PCR (qPCR; Schauss et al. 2009). Broadly, functional diversity is the representation of various aspects of overall soil microbial diversity. The Biolog‐Eco method is a useful tool to evaluate disturbances in soil microbial functional diversity and community due to different stresses (Lupwayi et al. 2009; Wang et al. 2010), and is thought to be one of the quickest and most effective methods available (Staddon et al. 1997). In addition, the activities of soil enzymes are sensitive indicators of the functional diversity of soil microbial communities due to their roles in soil biology (Tian and Shi 2014; Zaccardelli et al. 2013; Bending et al. 2002).

Moreover, Unlike the Biolog‐Eco method, which relies on the activities of those species that have adapted to rapid growth on simple substrates, soil enzyme activity is cultivated independently and to some extent represents the functioning of the entire microbial community. To date, few studies of the microbial effects of sugar alcohol amendments have employed both methods. Therefore, we utilized a combination of these two methods to compare functional changes induced by soil amendment with sugar alcohols. The objective of this study was to analyze community‐level physiological profile (CLPP) using the Biolog‐Eco method and enzymatic activities to identify and quantify specific effects of sugar alcohols on soil microbial functional diversity. These results will improve our understanding of the influence of sugar alcohols on soil fertility and quality.

## Experimental Procedures

### Experimental design

Soil (top 0–25 cm) was collected from the experimental plots of the Zhengzhou Fruit Research Institute, CAAS. The field had not been planted with crops in the last 5 years. The soil was classified as a silty loam, and its properties were as follows: 7.0 g organic matter/kg soil, 47.15 mg available P/kg soil, 127.2 mg available K/kg soil, 10.64 mg NH_4_^+^‐N/kg soil, 4.17 mg NO_3_^−^N/kg soil, and pH 7.62. Mannitol and sorbitol were not detected according to the method previously described by Mechri et al. (2015). After collection, the soil was homogenized, air‐dried for 48 h, sieved with a 2‐mm mesh to remove any plant tissue, grit, and soil animals, and then used for the experiments described below.

A 60‐day study was performed, and for each sugar alcohol (sorbitol and mannitol) concentration, a set of three replicate microcosms was prepared by transferring 300 g of soil (dry weight, DW) into jars. Each sample was artificially supplemented by spiking 15 mL of a sugar alcohol solution at calculated concentrations to produce final sugar alcohol concentrations of 0. 25, 0.5, and 1.0 g/kg DW soil (C0.25, C0.5, and C1, respectively). The control soil received an equivalent amount of distilled water. Each treatment was incubated at 25°C, and distilled water was added at regular intervals to maintain a water content of 60% of the maximum water holding capacity. For each microcosm, soil was taken at 60 days of incubation and stored at −20°C, as required for further analysis.

### Community‐level physiological profiles

Functional diversity of the soil microbial community was assessed using Biolog Eco Plates^™^ as described by Garau et al. (2007). The plate was composed of 96 wells containing a triplicate set of 31 carbon sources (ten carbohydrates, seven carboxylic acid (CA), four polymers, six amino acids, two phenolic compounds, and two amines) as well as three control wells with no carbon. Briefly, approximately 5 g of fresh soil was suspended in 50 mL of saline solution (0.85% NaCl, w/v) in a 250‐mL flask. After being shaken for 30 min (300 rpm) at 25°C, the suspensions were settled for 10 min. Subsequently, each suspension was diluted 100‐fold, and 150 *μ*L of the clear supernatant was inoculated directly into the Biolog plate, which was then incubated in the dark at 25°C for 7 days. Microbial development was monitored by reading the optical density (OD) at 590 nm every 24 h using a Biolog Microstation^™^ reader (Biolog., Hayward, CA, USA). The data collected were expressed as the following five parameters: average well color development (AWCD) for the metabolic activity of soil bacterial community, Shannon index (H’), Simpson index (D), substrate evenness (E), and substrate richness (S) at 96 h after addition of sugar alcohols.

### Soil enzyme activities

Invertase activity was measured according to the method previously described by Yao and Huang (2006) as follows: in a 150‐mL volumetric flask, 2 g of fresh soil was mixed with 15 mL of 8% sucrose, 5 mL of phosphate buffer (pH 5.5), and 5 drops of methylbenzene, followed by incubation at 37°C for 24 h. After filtration, 1 mL of filtrate and 3 mL of 3,5‐dinitrosalicylic acid (DNS) were added to react in a 50‐mL centrifuge tube, followed by incubation in a boiling bath for 5 min. Finally, the solution was diluted to 100 mL, and absorbance at 508 nm was measured using a spectrophotometer.

Urease activity was determined as described by Yao and Huang (2006). Briefly, 5 g of soil and 1 mL of methylbenzene were added to a 150‐mL conical flask. After 15 min, 10 mL of 10% urea and 20 mL of citrate buffer (pH 6.7) were mixed with the soil sample and then incubated at 37°C for 24 h. After filtration, 3 mL of filtrate was added to a 50‐mL volumetric flask and mixed with 4 mL of sodium phenoxide and 3 mL of sodium hypochlorite. After dilution to 50 mL and within 1 h, the absorbance at 578 nm was measured using a spectrophotometer.

Catalase activity was measured according to Li et al. (2008). Briefly, in a 150‐mL conical flask, 2 g of soil and 40 mL of distilled water were mixed with 5 mL of 0.3% H_2_O_2_. The flask was then sealed and shaken at 120 rpm for 20 min. To terminate the reaction, 5 mL of 1.5 mol/L H_2_SO_4_ was added to the flask. After filtration, 25 mL of filtrate was titrated with KMnO_4_.

Alkaline phosphatase activity was measured according to the method previously described by Guan (1986). Briefly, 2 g of soil was mixed with 20 mL of borate saline buffer (pH 9.6) and five drops of methylbenzene in a 150‐mL conical flask, and the mixture was incubated at 37°C for 24 h. Then, 40 mL of 3% aluminum sulfate solution was added to the flask. After filtration, 3 mL of filtrate and four drops of 2,6‐dibromoquinone‐4‐chloroimide were transferred to a 50‐mL volumetric flask and then diluted to 50 mL. The absorbance at 660 nm was determined using a spectrophotometer.

*β*‐Glucosidase activity was measured according to the method previously described by Eivazi and Tabatabai (1988). Briefly, 1 g of soil was weighed into 50‐mL glass vials, and 4 mL of phosphate buffer (pH = 6.0) and 1 mL of substrate (*p*‐nitrophenyl‐*β*‐d‐glucopyranoside) were added. The soil sample was then mixed thoroughly and incubated at 37°C for 1 h, after which 1 mL of 0.5‐mol/L CaCl_2_ and 4 mL Tris buffer (pH 12) were added. The resulting suspensions were filtered immediately and the absorbance at 400 nm was measured using a spectrophotometer.

*N*‐acetyl‐*β*‐d‐glucosaminidase activity was assayed using the method described by Ekenler and Tabatabai (2002). Briefly, 1 g of soil was weighed into 50‐mL glass vials, treated with 4 mL of 0.1 mol/L acetate buffer (pH 5.5) and 1 mL of 10‐mmol/L *p*‐nitrophenyl‐*N*‐acetyl‐*β*‐d‐glucosaminide as a substrate. The flask was stoppered, swirled to mix the contents, and incubated at 37°C. After 1 h of incubation, 1 mL of 0.5‐mol/L CaCl_2_ and 4 mL of 0.5‐mol/L NaOH were added to stop the reaction. The samples were filtered and the color intensity of the filtrate at 405 nm was measured using a spectrophotometer.

### Data analysis

The values in the figures and tables correspond to the average of triplicate data ± standard error (SE). The significance of differences between concentrations was tested by one‐way ANOVA using SPSS ver. 17 software (SPSS Inc., Chicago, IL, USA). Varimax rotated component matrix of carbon sources was conducted using SPSS ver. 17. Principal component analysis (PCA) of soil microbial CLPPs was performed with the Canoco software ver. 4.5 (Biometris., Wageningen, The Netherlands). Enzyme activities were calculated and the AWCD growth curve generated using SigmaPlot ver. 10 software (Systat Software Inc., San Jose, CA, USA).

## Results

### Sugar alcohols affect CLPPs

**Figure 1:**
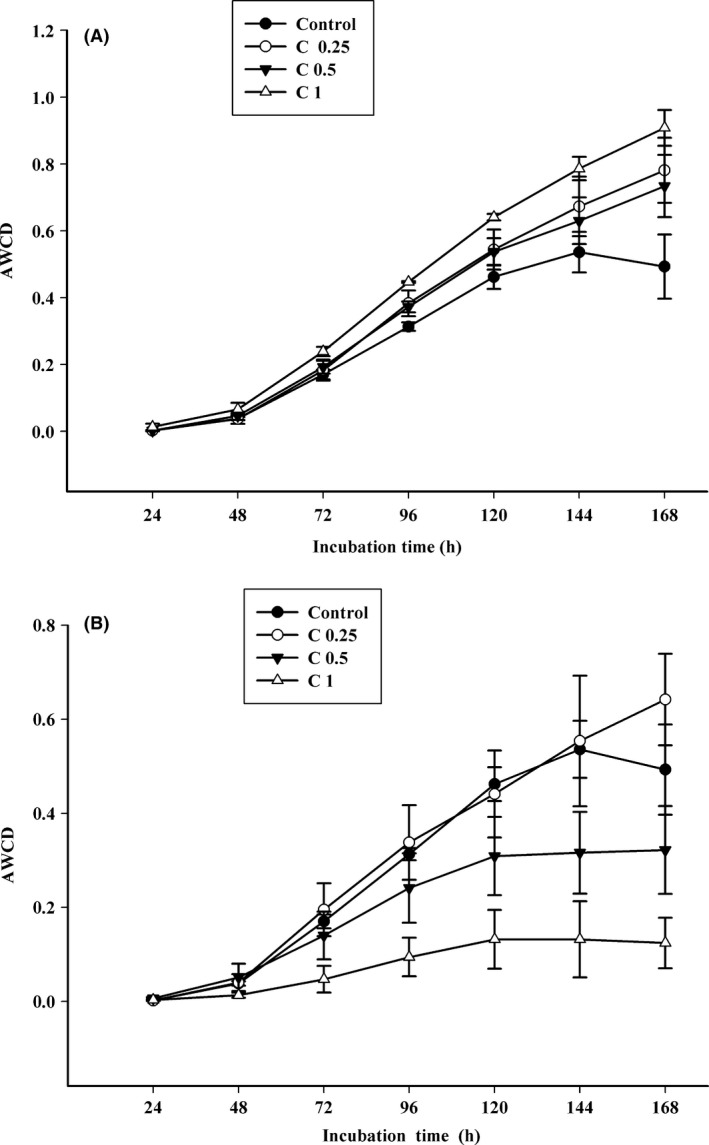
Variation in the average well color development of substrate utilization profiles of soil treated with (A) sorbitol and (B) mannitol. C0.25, C0.5, and C1 represent concentrations of 0.25, 0.5, and 1.0 g/kg dry weight soil after addition of sorbitol and mannitol, respectively. Vertical bars represent the standard error.

Average well color development (AWCD) was used as an indicator of soil microbial activity. Variation in AWCD with incubation time and addition of sugar alcohols was evaluated (Fig. 1). AWCD of the soil was almost zero after the first 24‐h incubation period, but gradually increased with incubation time. The addition of sorbitol caused an obvious increase in AWCD as compared to the control (Fig. 1A). In particular, the highest sorbitol concentration condition (C1) had the higher carbon utilization rate compared to other concentrations. However, as mannitol concentrations increased, the AWCD of all samples decreased; particularly, when the concentration of mannitol was 1.0 g/kg (C1), AWCD dropped to near zero during the incubation (Fig. 1B). The distinct AWCD decrease with mannitol addition revealed that mannitol has a suppressive effect on AWCD.

The effects of different concentrations of sugar alcohols on microbial functional diversity, as reflected by the Shannon diversity index, Simpson index, and substrate evenness and richness, were evaluated (Table 1). The Shannon diversity and Simpson indices of soils following sorbitol addition were slightly higher than the control, indicating few significant differences among the treatments. However, the Shannon diversity and Simpson indices of soils following mannitol addition (C1) were significantly lower than those for other treatments. Conversely, the substrate evenness for mannitol (C1) amendment showed a dramatic increase that was significantly higher than that with other treatments, whereas no significant differences were observed between the control and sorbitol. The substrate richness in the sorbitol addition treatments was higher than that for the control. Moreover, the increase was significantly higher with C1 treatment. Although the substrate richness was found to be significantly lower in mannitol treatment relative to the control, differences were also observed among different concentrations of the mannitol treatment.

**Table 1:** Shannon diversity index (H’), Simpson index (D), Substrate evenness (E), and Substrate richness (S) of the microbial functional diversity in sandy soils in relation to sugar alcohol treatment at 96 h after addition of sugar alcohols

Treatment	Shannon diversity index (H’)	Simpson index (D)	Substrate evenness (E)	Substrate richness (S)
Control	2.73 ± 0.08^ab^	0.92 ± 0.01^ab^	1.05 ± 0.01^b^	13.67 ± 1.2^abc^
Sorbitol				
C0.25	2.85 ± 0.08^a^	0.93 ± 0.01^ab^	1.02 ± 0.01^b^	16.67 ± 1.45^ab^
C0.5	2.84 ± 0.02^a^	0.93 ± 0^ab^	1.00 ± 0.01^b^	17.33 ± 0.88^ab^
C1	3.04 ± 0.03^a^	0.94 ± 0^a^	1.02 ± 0.01^b^	19.67 ± 0.33^a^
Mannitol				
C0.25	2.58 ± 0.22^abc^	0.90 ± 0.02^ab^	1.04 ± 0.04^b^	13.33 ± 3.28^bc^
C0.5	2.31 ± 0.32^bc^	0.86 ± 0.05^bc^	1.15 ± 0.07^b^	8.67 ± 2.91 ^cd^
C1	2.16 ± 0.06^c^	0.81 ± 0.03^c^	1.68 ± 0.22^a^	3.00 ± 1.15^d^

Data represent the mean of three replicates ± standard error. Different letters indicate significant difference within a column (*P < *0.05).

The use of six types of substrates (carbohydrates, amino acids, polymers, amines, phenolic compounds, and CAs) with sugar alcohol supplementation is listed in Table 2. Addition of sorbitol had positive effect on utilization of the substrate categories. Compared to the control, C0.5 and C1 significantly increased utilization of CAs by 112% and 128%, respectively. There was no significant difference in the utilization of substrates between mannitol addition and the control, except for C0.25 using amino acids.

**Table 2:** Average optical density of six types of substrates at 96 h after addition of sugar alcohols for 60 days

Treatment	Carbohydrates	Amino acids	Carboxylic acids	Polymers	Amines	Phenolic compounds
Control	0.35 ± 0.04^a^	0.35 ± 0.01^bc^	0.25 ± 0.02^bc^	0.51 ± 0.14^ab^	0.02 ± 0.02^a^	0.29 ± 0.12^ab^
Sorbitol						
C0.25	0.35 ± 0.03^a^	0.57 ± 0.12^ab^	0.34 ± 0.08^b^	0.6 ± 0.06^a^	0.06 ± 0.03^a^	0.20 ± 0.03^ab^
C0.5	0.39 ± 0.02^a^	0.38 ± 0.04^abc^	0.53 ± 0.04^a^	0.4 ± 0.03^ab^	0.05 ± 0.03^a^	0.48 ± 0.13^a^
C1	0.41 ± 0.02^a^	0.52 ± 0.03^ab^	0.63 ± 0.05^a^	0.39 ± 0.02^ab^	0.08 ± 0.07^a^	0.35 ± 0.05^a^
Mannitol						
C0.25	0.30 ± 0.08^a^	0.6 ± 0.1^a^	0.33 ± 0.04^b^	0.43 ± 0.07^ab^	0.00 ± 0^a^	0.37 ± 0.13^a^
C0.5	0.41 ± 0.05^a^	0.23 ± 0.08^c^	0.29 ± 0.09^b^	0.31 ± 0.06^b^	0.02 ± 0.01^a^	0.05 ± 0.02^b^
C1	0.09 ± 0.05^b^	0.2 ± 0.07^c^	0.10 ± 0.04^c^	0.05 ± 0.03^c^	0.01 ± 0.01^a^	0.01 ± 0.00^b^

Different letters indicate significant differences within a column (*P < *0.05). Data represent the mean of three replicates ± standard error.

**Figure 2:**
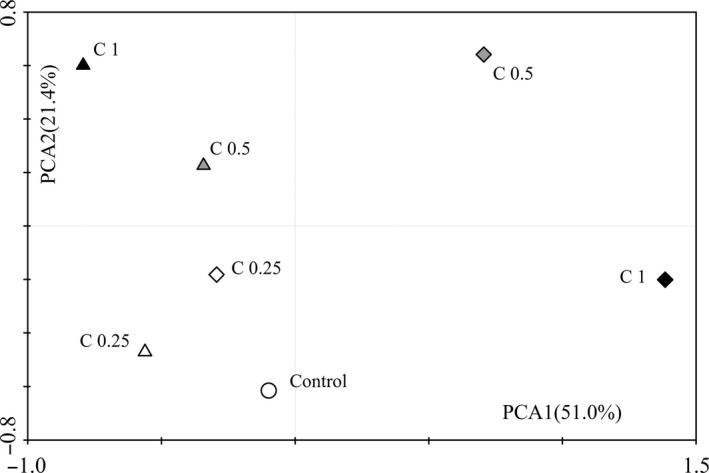
Principal component analysis of the soil microbial community‐level physiological profiles of the soils treated with sorbitol and mannitol. Triangle represents sorbitol treatment; diamond represents mannitol treatment; circle represents control.

Principal component analysis (PCA) of soil microbial CLPP revealed that addition of sugar alcohols changed the soil microbial community functional structure (Fig. 2). Applying PCA, we identified two principle components (PC1 and PC2) that accounted for 51.0% and 21.4% of the variation, respectively. PC1 and PC2 were plotted (Fig. 2), together accounting for 72.4% of the total variance. The principle component plot of the treatments showed clear separation, with the differences along the PC1 axis caused by sugar alcohols being larger than those along the PC2 axis. The substrate utilization patterns from medium and highest concentrations of mannitol amendment (C0.5 and C1) were located at the positive end of the PC1 axis, whereas most other treatments were located at the negative end. Moreover, the effects of medium and highest concentrations (C0.5 and C1) of sorbitol amendment were clearly different from the control along the PC2 axis. Meanwhile, the rotated component matrix (Table 3) identified the types of carbon sources with high loading (>0.75) for PC1 and PC2 as carbohydrates (CH), polymers (PM), and CA, along with sorbitol and mannitol (Yin et al. 2014; Dong et al. 2013).

**Table 3:** Varimax rotated component matrix of carbon sources

Carbon sources	Sort	PC1	PC2
*β*‐Methyl‐d‐Glucoside	CH	0.85	−0.18
d‐Galactonic Acid *γ*‐Lactone	CH	0.46	0.53
d‐Xylose	CH	0.57	0.59
i‐Erythritol	CH	0.91	−0.15
d‐Mannose	CH	0.33	0.53
*N*‐Acetyl‐d‐Glucosamine	CH	0.93	0.18
d‐Cellobiose	CH	0.85	−0.19
Glucose‐1‐Phosphate	CH	0.69	0.71
*α*‐d‐Lactose	CH	0.90	−0.25
d,l‐ *α*‐Glycerol	CH	−0.51	0.84
l‐Arginine	AA	0.57	−0.55
l‐Asparagine	AA	0.76	−0.53
l‐Phenylalanine	AA	0.72	−0.41
l‐Serine	AA	0.42	0.06
l‐Threonine	AA	0.80	0.11
Glycyl‐l‐Glutamic Acid	AA	−0.34	0.86
Pyruvic Acid Methyl Ester	CA	0.89	−0.19
d‐Galacturonic Acid	CA	0.56	0.13
*γ*‐Hydroxybutyric Acid	CA	−0.31	0.88
d‐Glucosaminic Acid	CA	0.58	0.26
Itaconic Acid	CA	0.94	−0.80
*α*‐Ketobutyric Acid	CA	0.84	0.46
d‐Malic Acid	CA	0.49	0.66
Tween 40	PM	0.90	0.04
Tween 80	PM	0.76	−0.31
*α*‐Cyclodextrin	PM	0.11	−0.31
Glycogen	PM	0.75	−0.15
Phenylethyl‐amine	AN	0.31	0.91
Putrescine	AN	0.62	0.14
2‐Hydroxy Benzoic Acid	PC	0.14	0.45
4‐Hydroxy Benzoic	PC	0.71	0.54

CH, carbohydrates; AA, amino acids; CA, carboxylic acids; PM, polymers; AN, amines; PC, phenolic compounds.

### Effects of sugar alcohols on soil enzyme activities

**Figure 3:**
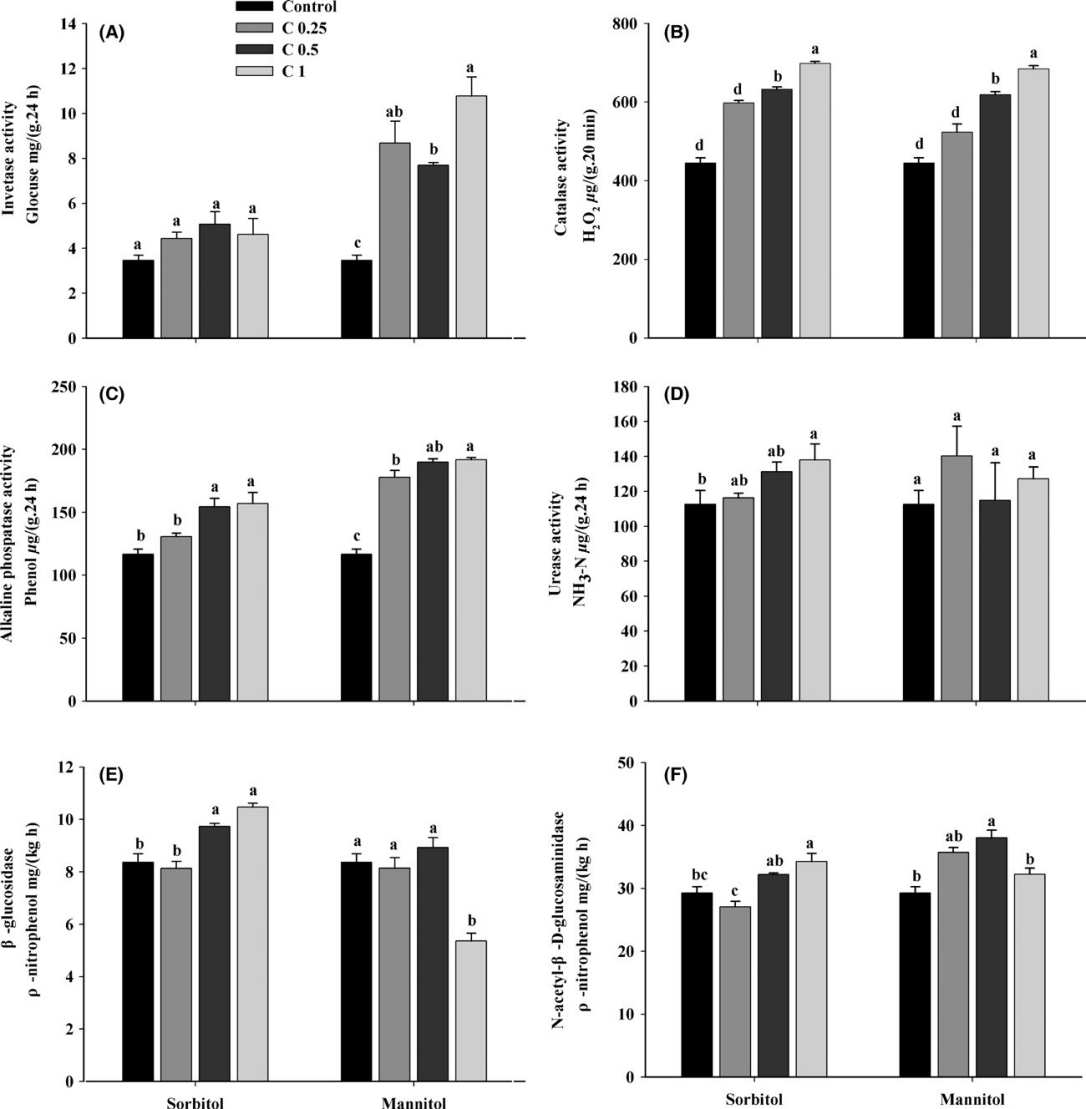
Soil enzyme activities of sorbitol‐ and mannitol‐treated soils. Soil (A) invertase, (B) calatase, (C) alkaline phosphatase, (D) urease, (E) *β*‐glucosidase and (F) *N*‐acetyl‐*β*‐d‐glucosaminidase activities. Values are mean ± SE, and letters denote significant differences among sugar alcohol concentrations (*P < *0.05).

The activities at 60 days of incubation were determined in the control and sugar‐ alcohol‐treated soils (Fig. 3). Invertase activity increased after the addition of sugar alcohols (Fig. 3A). However, invertase activity in sorbitol‐treated samples was slightly higher than that in the control, but no significant differences were found between the concentrations evaluated. Mannitol addition increased invertase activity significantly compared to the control.

The addition of sugar alcohols also caused significant effects on catalase activity (Fig. 3B). The average catalase activity in sorbitol and mannitol treatments increased by 44.44% and 36.78%, respectively, with maximum, significantly increased values of 56.85% and 53.75% at the highest concentration (C1), respectively. Furthermore, catalase stimulation was dependent on the sugar alcohol concentration, with the greatest stimulation during sorbitol and mannitol treatments observed at the highest treatment concentrations.

Similar to catalase activity, the activity of alkaline phosphatase was positively affected during treatments with sugar alcohols (Fig. 3C). When compared with the control, sorbitol and mannitol treatments on average increased alkaline phosphatase activity by 26.42% and 59.93%, respectively. The most pronounced increase in catalase activity in the sorbitol and mannitol treatments was observed primarily at the highest concentrations. Moreover, there were significant differences between the treatments and control.

The urease activity in the sorbitol‐treated samples was higher than that in the control, indicating that urease activity was stimulated by sorbitol addition (Fig. 3D). Moreover, the maximum urease activity occurred at the highest sorbitol concentrations, suggesting that stimulation was more pronounced at the highest sorbitol concentrations. However, urease activity was scarcely influenced by mannitol addition.

*β*‐Glucosidase activity was affected to some extent by sugar alcohols (Fig. 3E). When compared with the control, the *β*‐glucosidase activity in the sorbitol‐treated samples increased significantly by 16.37% and 28.84% at the medium and highest concentrations, respectively. However, compared with the control, addition of the highest concentration of mannitol significantly inhibited *β*‐glucosidase activity.

The addition of sugar alcohols exerted positive effects on *N*‐acetyl‐*β*‐d‐glucosaminidase activity (Fig. 3F). Compared with the control, treatment with the highest concentration of sorbitol significantly increased *N*‐acetyl‐*β*‐d‐glucosaminidase activity by 17.12%. The greatest increase in *N*‐acetyl‐*β*‐d‐glucosaminidase activity occurred following addition of the medium concentration of mannitol.

The correlation analysis (Table 4) showed that CAE, ALP, and URE activities were positively correlated with AWCD, S, and CA. There were also significant positive correlations of INE with S following sorbitol treatment. However, INE activities were negatively correlated with AWCD, H, D, S, and CH, and CAE was negatively correlated with S, PM, and PC after mannitol treatment.

**Table 4:** Correlation of enzyme activities with CLPP and carbon sources

	Sorbitol						Mannitol					
	INE	CAE	ALP	URE	GLU	NAG	INE	CAE	ALP	URE	GLU	NAG
AWCD	0.494	0.776^a^	0.632^b^	0.593^b^	0.407	−0.713^a^	−0.630^b^	−0.566	−0.386	−0.402	0.303	0.655^b^
H	0.484	0.712^a^	0.555	0.546	0.342	−0.690^b^	−0.577^b^	−0.498	−0.482	−0.512	0.174	0.546
D	0.356	0.717^a^	0.527	0.406	0.507	−0.655^b^	−0.612^b^	−0.565	−0.508	−0.527	0.263	0.648^b^
S	0.596^a^	0.816^a^	0.657^a^	0.584^b^	0.427	−0.643^b^	−0.667^b^	−0.637^b^	−0.5	−0.348	0.274	0.657^b^
E	−0.515	−0.61	0.548	−0.34	−0.478	0.175	0.676^b^	0.695^b^	0.426	0.167	−0.423	−0.792^a^
CH	0.055	0.51	0.43	0.332	0.221	−0.643^b^	−0.668^b^	−0.396	−0.276	−0.466	0.507	0.725^a^
AA	0.284	0.406	0.242	0.212	0.354	−0.533	−0.24	−0.445	−0.153	0.124	0.114	0.235
PM	−0.356	−0.378	−0.368	−0.169	−0.086	0.142	−0.722^a^	−0.77^a^	−0.538	−0.097	0.292	0.781^a^
AN	0.023	0.365	0.44	0.227	0.343	−0.376	−0.198	−0.014	−0.095	−0.15	0.21	0.085
PC	0.41	0.224	0.281	0.026	0.257	0.177	−0.377	−0.581^b^	−0.478	−0.16	0.111	0.315
CA	0.379	0.839^a^	0.826^a^	0.687^b^	0.446	−0.525	0.748^a^	0.822^a^	0.734^a^	0.1	−0.081	−0.638^b^

Enzyme activities: INE, invertase; CAE, catalase; ALP, alkaline phosphatase; URE, urease; GLU, *β*‐glucosidase; NAG, *N*‐acetyl‐*β*‐d‐glucosaminidase.

a Correlation is significant at the 0.01 level.

b Correlation is significant at the 0.05 level.

## Discussion

Soil amendment with organic substances is a major driving force for changes in soil microbial community composition (Ai et al. 2012; Bowles et al. 2014), and these changes can be inferred from CLPP data (Sprocati et al. 2014). In this study, the type of organic substance used as a soil amendment was identified as an impact factor for CLPP. Soil amendment with sorbitol had a strong influence on CLPP and microbial functional diversity parameters, which, except for substrate evenness, were higher than the control (Table 1). Similar results showing carbon source effects on soil microbial community composition and function have been reported (Gomez et al. 2006; Hu et al. 2011; Cheng et al. 2014). However, in this study, a contrary trend was observed after addition of mannitol, particularly at the medium and highest concentrations. These results indicate that different types and concentrations of sugar alcohols affect CLPP differently. Furthermore, soil microbial community with sorbitol and mannitol treatment did not show a preference for the 31 carbon sources (Table 3). PCA clearly revealed differences in the effects on soil microbial community function. Meanwhile, by using a rotated component matrix, the results clearly showed that the utilization of carbohydrates, polymers, and CAs affected soil microbial community structure and function, indicating that these three kinds of carbon sources likely cause differentiation of soil microbial communities.

The investigation of enzyme activities is important as they indicate the potential of a soil to carry out biochemical reactions upon addition of organic matter (Ladd 1985), and are related to nutrient cycling and organic matter dynamics in soil (Burns 1982; Dick et al. 1997; Pascual et al. 1998). Invertase, *β*‐glucosidase and *N*‐acetyl‐*β*‐d‐glucosaminidase activities are highly sensitive to substrate availability (Sinsabaugh et al. 1993), and are effective indicators of microbial activity (Kiss et al. 1972; Turner et al. 2002; Ekenler and Tabatabai 2002). Ureases catalyze the hydrolysis of organic to inorganic nitrogen. Alkaline phosphatases catalyze the hydrolysis of organic phosphorus compounds to phosphates (Wright and Reddy 2001). Moreover, catalases are associated with soil respiratory intensity and microbial activity, and so reflect the soil microbial processes to some extent. The levels of enzymes detected in sugar‐ alcohol‐treated soils were commonly higher compared with the control (Fig. 3), as has been reported previously (Jones and Murphy 2007; Hopkins et al. 2008). It might be speculated that sugar alcohols act as an organic substrate to stimulate enzyme release as well as increase microbial biomass and activity (Kuzyakov and Bol 2006). Furthermore, this study demonstrated that the type and concentration of sorbitol and mannitol have different effects on soil enzyme activities. This is not surprising given that some neutral sugars can induce respiration and are strongly correlated with soil microbial biomass content at proper concentrations (Blagodatskaya and Kuzyakov 2008; Dungait et al. 2013). In addition, *β*‐glucosidase activity following treatment with the highest concentration of mannitol was decreased (Fig. 3E). This interesting result was supported by Luxhøi et al. (2002) who reported that addition of organic matter with a high lignin content inhibited *β*‐glucosidase activity. However, the *β*‐glucosidase activity showed the opposite trend after addition of sorbitol. Moscatelli et al. (2012) and Roldán et al. (2005) reported that *β*‐glucosidase activities are positively correlated with C availability in soils treated with organic matter. In this study, invertase and urease activities were not affected by the addition of sorbitol or mannitol (Fig. 3), although it is commonly understood that the activities of invertase and urease are related to soil organic carbon. Thus, other factors may regulate enzyme activity. Moreover, in this study, the use of air‐dried soils and the selected incubation periods could have affected the assessment of microbial community and soil enzyme activities as affected by the quality of sugar alcohol substrates, in which only potential and not actual activities were measured (Yao et al. 2006; Lee et al. 2007; Nannipieri et al. 2012).

In this study, correlation analysis showed a positive relationship between microbial functional diversity indices (with the exception of substrate evenness) and catalase activity, following amendment with sorbitol. This indicated that the Biolog cultivable community was an important contributor to catalase production following sorbitol supplementation. Conversely, following mannitol addition, invertase activities were negatively correlated with microbial functional diversity indices (with the exception of substrate evenness). A similar result was obtained in a previous study in which enzymes activities were significantly negatively related to H` (Yin et al. 2014). Thus, the correlation analysis indicated that microbial community composition displays no unique variation in potential enzyme activities. The size and physiology of the microbial community also directly affects enzyme activity in soil (Schimel and Gulledge 1998; Wall and Moore 1999). Therefore, it was essential that the Biolog‐Eco plate method and enzyme activity assays be utilized in parallel to assess aerobic, functional microbial diversity.

## Conclusions

The response of a soil microbial community to amendment with sugar alcohols was investigated through a single application at three levels. Our results suggest that a higher concentration of sorbitol can increase microbial functional diversity and enzyme activities. Some enzyme activities were also enhanced by mannitol addition, although there were obvious inhibitory effects on microbial functional diversity with increasing mannitol concentrations. This study clearly demonstrates that soil enzyme activities and microbial functional diversity (assayed by Biolog) have different susceptibilities to sorbitol and mannitol, revealing selective pressure on soil microbial community function during the cultivable period. Future studies will seek to more clearly define these relations, helping to improve our understanding of the effects of sugar alcohols on soil ecosystems.

## Conflict of Interest

None declared.
